# Rediscovering *Citrus lumia* ‘Pyriformis’: Phytochemical Profile and Multifunctional Properties of Its Fresh Juice

**DOI:** 10.3390/foods14233997

**Published:** 2025-11-21

**Authors:** Antonella Smeriglio, Annarita La Neve, Marta Mangano, Martina Imbesi, Laura Cornara, Domenico Trombetta

**Affiliations:** 1 Department of Chemical, Biological, Pharmaceutical and Environmental Sciences, University of Messina, Viale Ferdinando Stagno d’Alcontres 31, 98166 Messina, Italy; annarita.laneve@studenti.unime.it (A.L.N.); marta.mangano@studenti.unime.it (M.M.); martina.imbesi@studenti.unime.it (M.I.); domenico.trombetta@unime.it (D.T.); 2 Department of Earth, Environment and Life Sciences (DISTAV), University of Genova, Corso Europa 26, 16132 Genova, Italy; laura.cornara@unige.it

**Keywords:** *Citrus lumia*, juice, polyphenols, antioxidant activity, anti-inflammatory activity, wound-healing activity, ascorbic acid, citric acid, functional foods, mediterranean biodiversity

## Abstract

This study provides the first comprehensive chemical and biological profiling of *Citrus lumia* Risso & Poit. var. ‘Pyriformis’, a rare Mediterranean *Citrus* variety with unexplored nutraceutical potential. The fresh juice (CLPJ) showed a distinctive phytochemical composition, with 38.8 ± 0.99 mg gallic acid equivalents/100 mL of total phenols and 25.96 ± 2.37 mg rutin equivalents/100 mL of flavonoids. High-performance liquid chromatography coupled with diode-array detection (HPLC-DAD) quantification revealed high levels of organic acids, including ascorbic acid (0.34 g/L) and citric acid (34.6 g/L). Liquid chromatography coupled with diode-array detection and electrospray ionization tandem mass spectrometry (LC-DAD-ESI-MS/MS) enabled the annotation of 28 polyphenolic constituents, featuring glycosylated flavanones and several uncommon flavonols and acylglycosidic derivatives whose structural patterns are typical of primitive *Citrus* lineages and largely absent in commercial cultivars. Functionally, CLPJ exhibited multi-target antioxidant and anti-inflammatory activities and promoted epithelial repair in Caco-2 cells without cytotoxic effects. Overall, the juice displays a distinctive chemotaxonomic fingerprint and promising multifunctional properties, supporting its potential as a functional food ingredient and contributing to the valorization of minor Mediterranean *Citrus* biodiversity.

## 1. Introduction

Lifestyle changes and the growing awareness of the relationship between diet and health have strengthened the importance of functional foods, particularly fruits and vegetables, as fundamental tools for disease prevention [1,2]. Among these, *Citrus* fruits (family Rutaceae) occupy a prominent role in human nutrition, not only for their pleasant sensory properties but also for their richness in bioactive compounds with proven antioxidant, anti-inflammatory, and cytoprotective effects [3,4].

Although a few species—mainly oranges, mandarins, lemons, and grapefruits—dominate global production, the *Citrus* genus encompasses a remarkable taxonomic and genetic variability, including numerous minor species, cultivars, and hybrids [5,6]. These taxa represent an invaluable reservoir of chemical diversity and play a crucial role in the preservation of Mediterranean agro-biodiversity [7]. Rediscovering and characterizing neglected or underutilized varieties could therefore provide new sources of functional ingredients and contribute to the valorization of traditional crops.

Among the lesser-known species, *Citrus lumia* Risso, an ancient Mediterranean taxon historically cultivated in Sicily, stands out for its peculiar morphology and aromatic properties. Once appreciated for both alimentary and traditional uses, this species has almost disappeared from commercial markets. In southern Italy, particularly in the Agrigento area, *C. lumia* was used as a natural air freshener, digestive aid, and calming remedy. The fruit is characterized by its ovoid, mamillate shape with a pronounced umbo and by an unusually sweet juice compared to other *Citrus* species [8].

Over the last few years, our research group has systematically investigated *C. lumia* through a multidisciplinary approach combining phytochemical and biological analyses. The essential oil, rich in D-limonene, β-pinene, and γ-terpinene, displayed strong antioxidant, anti-inflammatory, and acetylcholinesterase-inhibitory activities [9]. The albedo showed a high polyphenolic content (eriocitrin, hesperidin) and cytoprotective effects in lymphocytes exposed to oxidative stress [10]. The seeds contained relevant amounts of tocopherols, flavonoids, and phenolic acids, exhibiting potent antioxidant and anti-inflammatory properties in both cell-free and peripheral blood mononuclear cells [11]. The fresh juice revealed a complex polyphenolic profile and high levels of ascorbic acid, associated with significant antioxidant, anti-inflammatory, and anti-angiogenic activities without cytotoxic effects [12].

*Citrus* juices, widely distributed and globally consumed, provide a natural balance of water, vitamins, minerals, sugars, carotenoids, fibers, and organic acids, along with a pleasant and refreshing taste [13]. Although there is consensus regarding the protective effects of fruit and vegetable consumption, evidence supporting the health benefits of fruit juices remains more controversial, mainly due to their lower fiber content and higher caloric density compared to fresh fruits. However, 100% *Citrus* juices—free from additives and sweeteners and labeled as such by the U.S. FDA—retain a substantial portion of the original nutrients and bioactive compounds, such as ascorbic acid and polyphenols, both known for their beneficial effects on human health [13]. In addition to their nutritional relevance, juices from commonly consumed *Citrus* species such as *C. sinensis*, *C. paradisi*, *C. reticulata*, and *C. limon* are known to contain substantial amounts of vitamin C, phenolic acids, and glycosylated flavanones, which contribute to their antioxidant, anti-inflammatory, and cytoprotective properties. Several studies have shown that these juices can modulate oxidative stress, inflammation, and epithelial barrier function, largely through the activity of hesperidin, eriocitrin, naringin, and related metabolites. This established functional profile provides a useful reference framework for positioning the less-explored *C. lumia* accessions within the broader context of *Citrus*-derived health-promoting matrices [3,4].

Building upon this evidence, the present study focuses for the first time on the pear-shaped variety *C. lumia* Risso & Poit. var. ‘Pyriformis’, a rare cultivar so far investigated only for its essential oil composition [14]. This specific accession exhibits distinctive morphological traits compared with the commonly described *C. lumia* phenotype, yet its phytochemical and biological properties remain completely unexplored. For this reason, the present study was conceived to provide the first integrated characterization of its fresh juice, encompassing comprehensive phytochemical profiling, evaluation of its antioxidant and anti-inflammatory potential through complementary in vitro assays, and assessment of its cytocompatibility and ability to promote epithelial repair in intestinal (Caco-2) cells. Particular attention is given to its potential role in promoting epithelial regeneration and maintaining intestinal barrier integrity—key targets in the prevention and management of chronic inflammatory bowel conditions.

## 2. Materials and Methods

### 2.1. Plant Material and Juice Processing

The fruits of *C. lumia* Risso & Poit. var. ‘Pyriformis’ were kindly provided by the Hanbury Botanical Gardens of La Mortola, Ventimiglia (Italy). As all plant species cultivated and distributed by accredited botanical gardens are taxonomically certified and officially registered within their internal collections. Furthermore, the botanical identification was confirmed by the curator of the Hanbury Botanical Gardens. A total of 5 fully ripe fruits were collected in November 2024 from a single adult tree preserved within the historical *Citrus* collection. The collection site is located at coordinates 43.786° N, 7.602° E; altitude 82 m a.s.l. Fruits were selected based on uniform peel color, firmness, and absence of defects and shipped the same day to laboratory via priority air transport, where they were appropriately processed, ensuring minimal degradation of thermolabile or oxidation-prone constituents.

*C. lumia* ‘Pyriformis’ Juice (CLPJ) was obtained by manual squeezing and subsequently centrifuged at 3500× *g* for 15 min at 4 °C (Neya 10R, REMI, Mumbai, India). The supernatant, with a density of 1.06 g/cm^3^ at 20 °C, was collected, filtered through a 0.45 µm nylon disc syringe filter, and stored at −80 °C until analysis. For spectrophotometric analyses, the juice was suitably diluted in deionized water., while for HPLC analyses, it was diluted in Milli-Q water and filtered through a 0.22 µm nylon disc filter.

### 2.2. Chemicals

All chemicals used in this study were ACS reagent grade, whereas chromatographic solvents and analytical standards were of LC–MS grade. Unless otherwise stated, all reagents, inorganic salts, acids, bases, buffer components, chromogenic substrates, cell culture additives, and LC–MS grade acetonitrile and formic acid were purchased from Merck KGaA (Darmstadt, Germany). Polyphenolic standards were purchased from both Merck KGaA (Darmstadt, Germany) and Extrasynthase (Genay, France). Milli-Q grade water was obtained using a Direct-Q^®^ 3 UV water purification system equipped with integrated UV lamp and final 0.22 µm membrane filter (Millipore, Merck KGaA, Darmstadt, Germany).

### 2.3. Phytochemical Analyses

#### 2.3.1. Total Phenols

The total phenolic content was quantified according to the procedure described by Ingegneri et al. [15], using the Folin–Ciocalteu colorimetric assay. In brief, aliquots of CLPJ (31.25–500 mg/mL) were combined with Folin–Ciocalteu reagent and distilled water, followed by the addition of sodium carbonate after a short pre-incubation. The reaction mixtures were maintained in the dark at room temperature for 1 h and the absorbance was then recorded at 785 nm using a UV–Vis microplate reader (Multiskan™ GO, Thermo Scientific, Waltham, MA, USA). Calibration was performed with gallic acid (75–600 μg/mL, R^2^ = 0.9997), and results were expressed as mg gallic acid equivalents (GAE)/100 mL of juice.

#### 2.3.2. Total Flavonoids

The total flavonoid content was assessed following the colorimetric procedure reported by Smeriglio et al. [12]. CLPJ samples (31.25–500 mg/mL), previously diluted in distilled water, were reacted with sodium nitrite, aluminium chloride, and sodium hydroxide according to the referenced method. After the final incubation, absorbance was recorded at 510 nm using a UV–Vis spectrophotometer (Shimadzu UV-1601, Kyoto, Japan). Rutin (125–1000 μg/mL, R^2^ = 0.9998) was used for calibration, and the results were expressed as milligrams of rutin equivalents (RE)/100 mL of juice.

#### 2.3.3. Flavan-3-ols

Flavan-3-ols were quantified according to the procedure described by Ingegneri et al. [15]. CLPJ samples, previously acidified and passed through pre-conditioned C18 cartridges, were eluted with methanol and reacted with a methanolic vanillin solution followed by hydrochloric acid, as detailed in the referenced method. After incubation in the dark, absorbance was measured at 500 nm using the UV–Vis spectrophotometer described in Section 2.3.2. Calibration was performed with catechin (125–500 μg/mL, R^2^ = 0.9997), and results were expressed as mg catechin equivalents (CE)/100 mL of juice.

#### 2.3.4. Proanthocyanidins

Proanthocyanidins were quantified following the procedure reported by Ingegneri et al. [15]. Acidified CLPJ samples were loaded onto pre-conditioned C18 cartridges, and the retained fraction was eluted with methanol and transferred into ethanol. The eluates were then reacted with an iron(II) sulfate solution in hydrochloric acid and heated under reflux, according to the referenced method. After cooling, absorbance was recorded at 550 nm, subtracting the value of a control sample maintained on ice. Proanthocyanidin levels were calculated from the amount of cyanidin formed, using the molar extinction coefficient of cyanidin chloride (ε = 34,700), and expressed as mg cyanidin chloride equivalents (CcE)/100 mL of juice.

#### 2.3.5. Liquid Chromatography Coupled with Diode-Array Detection and Electrospray Ionization Tandem Mass Spectrometry (LC-DAD-ESI-MS/MS)

The polyphenolic profile of CLPJ was evaluated by liquid chromatography coupled with diode-array detection and electrospray ionization tandem mass spectrometry, following the analytical approach reported by Smeriglio et al. [12] with some modifications. Separation was carried out at 25 °C on a Luna Omega PS C18 column (150 × 2.1 mm, 5 µm; Phenomenex, Torrance, CA, USA) using water with 0.1% formic acid (solvent A) and acetonitrile (solvent B). The elution was performed with a multistep gradient ranging from 0% to 60% B over 81 min, followed by a return to initial conditions for column re-equilibration. The injection volume was 5 µL. UV–Vis data were acquired between 190 and 600 nm, and chromatograms were examined at 260, 292, 330 and 370 nm to monitor the main classes of phenolic compounds. Mass spectrometric analyses were conducted on an Agilent 6320 ion trap system (Agilent Technologies, Santa Clara, CA, USA) operated in both positive and negative ESI modes. The instrumental settings included a capillary voltage of 3.5 kV, nitrogen nebulization at 40 psi, drying gas at 350 °C with a flow of 9 L/min, and a skimmer voltage of 40 V. Spectra were recorded in full-scan mode over *m*/*z* 90–1000. The excitation amplitude applied in the ion trap during CID-induced MS/MS fragmentation was set to 1.2 V. Chromatographic and spectral data were processed using Agilent ChemStation (v. B.01.03) and Trap Control (v. 6.2). Compound annotation was based on retention behavior, UV–Vis absorption patterns, and mass spectral features. Instrument performance was monitored through replicate injections (*n* = 3) of the same sample, and retention time stability remained within acceptable limits (variation < 2%). For major constituents for which authentic standards were available, identification was confirmed by comparison with reference spectra and retention times. All other constituents were classified as putatively identified through comparison with literature data and open-access spectral databases (e.g., SpectraBase^®^, PhytoHub, ReSpect, MassBank, PubChem). Relative abundance in total ion chromatograms was expressed as ion peak intensity.

#### 2.3.6. Determination Ascorbic and Citric Acid Contents by High-Performance Liquid Chromatography Coupled with Diode-Array Detection (HPLC-DAD)

Ascorbic and citric acids in CLPJ were quantified by high-performance liquid chromatography coupled with diode-array detection (Agilent Technologies, Santa Clara, CA, USA). Aliquots of juice were diluted in the mobile phase to obtain final concentrations of 12 mg/mL for ascorbic acid and 5 mg/mL for citric acid, filtered through 0.22 μm nylon membranes, and injected into the chromatographic system. Analyses were conducted on a 5 μm Eclipse Plus C18 column (150 × 4.6 mm; Phenomenex, Torrance, CA, USA) equilibrated at 25 °C and eluted isocratically with 0.025% trifluoroacetic acid at a flow rate of 0.8 mL/min. The injection volume was 20 μL. UV–Vis monitoring was performed across 190–400 nm, with quantification based on chromatograms recorded at 245 nm for ascorbic acid and 210 nm for citric acid. Identification was verified by matching retention times and absorption spectra with those of certified reference standards (purity ≥ 99%). The HPLC-DAD method was validated by assessing linearity, precision, sensitivity, and accuracy following standard analytical criteria (Table 1). Calibration curves for ascorbic and citric acids were prepared across six concentration levels within the respective working ranges. Linearity was evaluated by regression analysis, while method sensitivity was assessed by calculating the limits of detection (LOD) and quantification (LOQ) according to the 3.3σ/slope and 10σ/slope criteria. Intra-day and inter-day precision were determined at three concentration levels (*n* = 6), and accuracy was verified through recovery experiments performed by spiking known amounts of each analyte into the matrix.

To minimize ascorbic acid oxidation during sample preparation and analysis, all solutions were freshly prepared, protected from light, and kept on ice. The autosampler, equipped with a Peltier cooling system, was maintained at 4 °C throughout the analytical sequence to ensure sample stability.

### 2.4. In Vitro Cell-Free Biological Assays

#### 2.4.1. 2,2-Diphenyl-1-picrylhydrazyl (DPPH) Assay

The DPPH radical scavenging activity of CLPJ was evaluated according to the procedure reported by Ingegneri et al. [15]. Diluted samples (2–24 mg/mL) were combined with a freshly prepared methanolic solution of DPPH (1 mM) and kept in the dark for 20 min. The decrease in absorbance was recorded at 517 nm using the microplate reader described in Section 2.3.1, with methanol as the blank. Trolox (2–20 μg/mL, R^2^ = 0.9998) was used as the reference antioxidant.

#### 2.4.2. Ferric Reducing Antioxidant Power (FRAP)

The FRAP of CLPJ was determined according to the procedure described by Ingegneri et al. [15]. Aliquots of the sample (1–12 mg/mL) were added to freshly prepared FRAP reagent—composed of acetate buffer, TPTZ solution, and FeCl_3_ and equilibrated at 37 °C—and the mixture was allowed to react for 4 min in the dark at room temperature. Absorbance was then measured at 593 nm using the plate reader and blank conditions reported in Section 2.3.1. Trolox (1–10 μg/mL; R^2^ = 0.9996) served as the calibration standard.

#### 2.4.3. Trolox Equivalent Antioxidant Capacity (TEAC)

The TEAC of CLPJ was determined using the ABTS^+•^ assay as described by Ingegneri et al. [15]. The ABTS^+•^ radical cation was generated by reacting ABTS with potassium persulfate and allowing the mixture to stand in the dark for 12 h at room temperature; the solution was then diluted to obtain an absorbance of 0.7 at 734 nm and used within 4 h. Aliquots of CLPJ (1–12 mg/mL) were mixed with the diluted radical solution and incubated for 6 min at room temperature. Absorbance was measured at 734 nm using the same plate reader and blank reported in Section 2.3.1. Trolox (1–10 μg/mL; R^2^ = 0.9998) was used as the reference antioxidant for calibration.

#### 2.4.4. The Oxygen Radical Absorbance Capacity (ORAC)

The ORAC of CLPJ was determined according to the method described by Ingegneri et al. [15]. Diluted samples (0.25–4 mg/mL) were combined with a freshly prepared fluorescein solution and incubated at 37 °C for 15 min. The reaction was initiated by adding AAPH, and fluorescence decay was monitored every 30 s for 90 min at excitation and emission wavelengths of 485 and 520 nm, respectively, using a Varioskan™ LUX multimode plate reader (Thermo Fisher Scientific, Waltham, MA, USA). Antioxidant capacity was calculated from the area under the fluorescence decay curve (AUC) and expressed relative to Trolox (0.25–2 μg/mL; R^2^ = 0.9999).

#### 2.4.5. Iron-Chelating Activity (ICA)

The metal-chelating capacity of CLPJ was assessed through the ferrozine assay following the procedure reported by Smeriglio et al. [10], with slight adaptations. CLPJ samples (1.25–20 mg/mL) were mixed with a ferrous chloride solution and allowed to react for 5 min at room temperature. The chromogenic reagent ferrozine was then added, and the mixture was incubated for a further 10 min. Absorbance was measured at 562 nm using the plate reader specified in Section 2.3.1. Ethylenediaminetetraacetic acid (EDTA, 50–400 μg/mL; R^2^ = 0.9995) served as the reference standard. Chelating activity was inversely proportional to the intensity of the ferrozine–Fe^2+^ complex formed.

#### 2.4.6. β-Carotene Bleaching (BCB)

The BCB assay was performed according to the procedure described by Smeriglio et al. [16]. CLPJ samples (0.125–2 mg/mL), butylated hydroxytoluene (BHT, 1 mg/mL) as the reference antioxidant, or deionized water as the blank were mixed with a freshly prepared β-carotene–linoleic acid emulsion containing β-carotene, linoleic acid, and Tween-40. The mixtures were incubated at 50 °C for 2 h to promote linoleic acid oxidation and the subsequent degradation of β-carotene. Absorbance was monitored every 20 min at 470 nm using the plate reader specified in Section 2.3.1. An emulsion lacking β-carotene was used as a negative control to correct for non-specific turbidity. Antioxidant activity was expressed as the ability of the sample to inhibit or delay β-carotene discoloration relative to the control.

#### 2.4.7. Albumin Denaturation Assay (ADA)

The ability of CLPJ to inhibit heat-induced bovine serum albumin (BSA) denaturation was evaluated according to the procedure described by Ingegneri et al. [15]. CLPJ samples (0.5–1.2 mg/mL) were mixed with a 0.4% BSA solution and PBS (pH 5.3). Sodium diclofenac (3–24 μg/mL; R^2^ = 0.9998) was used as the reference anti-inflammatory compound. Reaction mixtures were incubated at 70 °C for 30 min in a thermostatic water bath under gentle agitation, and absorbance was measured at 595 nm using the plate reader specified in Section 2.3.1. Readings obtained at time zero served as the baseline. A reduction in absorbance relative to the control indicated inhibition of protein denaturation.

#### 2.4.8. Protease Inhibition Assay (PIA)

Protease inhibitory activity was assessed using the trypsin–casein assay described by Ingegneri et al. [15]. CLPJ samples (1.25–10 mg/mL) or sodium diclofenac (5–80 μg/mL; R^2^ = 0.9997), used as the reference inhibitor, were incubated with trypsin in Tris-HCl buffer (pH 7.5) and subsequently mixed with a casein solution. The mixtures were maintained at 37 °C for 20 min to allow enzymatic hydrolysis. The reaction was terminated by adding perchloric acid to precipitate undigested proteins, followed by centrifugation at 3500× *g* for 10 min. The absorbance of the resulting supernatant, containing soluble peptides, was measured at 280 nm using the spectrophotometer reported in Section 2.3.2. Protease inhibition was calculated as the percentage decrease in absorbance compared with the enzymatic control.

### 2.5. Cell-Based Assays

#### 2.5.1. Cytotoxicity Assessment

Cytotoxicity was evaluated on human epithelial colorectal adenocarcinoma cell line Caco-2 (ATCC^®^ HTB-37™, Lot No. 70057475), purchased from the American Type Culture Collection (ATCC, Manassas, VA, USA) using the MTT colorimetric assay. Cells were maintained in DMEM supplemented with 10% FBS, 1% NEAA, and 1% penicillin–streptomycin and used up to passage 25. A total of 1 × 10^5^ cells/well (100 μL; 1 × 10^6^ cells/mL) was seeded into 96-well plates and allowed to adhere for 24–48 h at 37 °C in a humidified 5% CO_2_ atmosphere. After attachment, the medium was replaced with CLPJ solutions prepared in DMEM to achieve final concentrations of 0.04–20 mg/mL; complete DMEM served as the negative control. Following 24 h of treatment, the medium was removed, and cells were incubated with MTT solution (0.25 mg/mL in DMEM) for 4 h. The supernatant was then discarded, cells were washed with PBS, and formazan crystals were solubilized with acidified isopropanol. Absorbance was recorded at 570 nm using the plate reader specified in Section 2.3.1. Cell viability was expressed as a percentage relative to untreated controls.

#### 2.5.2. Scratch Assay

The wound-healing properties of CLPJ were assessed using a scratch assay on Caco-2 monolayers. Cells at passage 25 were cultured in DMEM supplemented with 10% FBS and penicillin–streptomycin and seeded in 24-well plates at 5 × 10^4^ cells/well (500 μL). After incubation for 72–96 h at 37 °C and 5% CO_2_, confluent monolayers were scratched with a sterile 200-μL pipette tip. Detached cells were removed by washing with PBS, and 500 μL of treatment solutions prepared in complete DMEM were added: sodium hyaluronate (0.1%) as the positive control, complete DMEM as the negative control, and CLPJ at 0.782–12.5 mg/mL. Plates were further incubated under standard culture conditions, and wound images were acquired at 0, 24 and 48 h (magnification: 10×) using an inverted phase-contrast microscope equipped with a digital camera (Leica DMi1, FLEXACAM C1, 12 MP; Leica Camera AG, Wetzlar, Germany). Wound closure was quantified using Capture 2.4 software (New York Microscope Company, Hicksville, NY, USA) and expressed relative to the wound area at time zero.

### 2.6. Statistical Analysis

All in vitro experiments—both cell-free and cell-based—were performed in three independent runs, each carried out in triplicate (*n* = 3). Statistical differences were evaluated by one-way ANOVA, and post hoc comparisons were conducted using the Student–Newman–Keuls test. Analyses were performed with SigmaPlot 12.0 (Systat Software Inc., San Jose, CA, USA), and significance was accepted at *p* < 0.05.

## 3. Results

### 3.1. Phytochemical Screening and LC-DAD-ESI-MS/MS Analyses

The preliminary phytochemical screening of CLPJ revealed a complex and diversified phenolic composition, with flavonoids representing the major bioactive fraction of the plant complex. As reported in Table 2, the total phenolic content, determined by the Folin–Ciocalteu method, was 38.80 ± 0.99 mg GAE/100 mL. The total flavonoid content, measured by the aluminum chloride colorimetric assay, was 25.96 ± 2.37 mg RE/100 mL, corresponding to approximately two-thirds of the total phenolic fraction.

**Figure 1:**
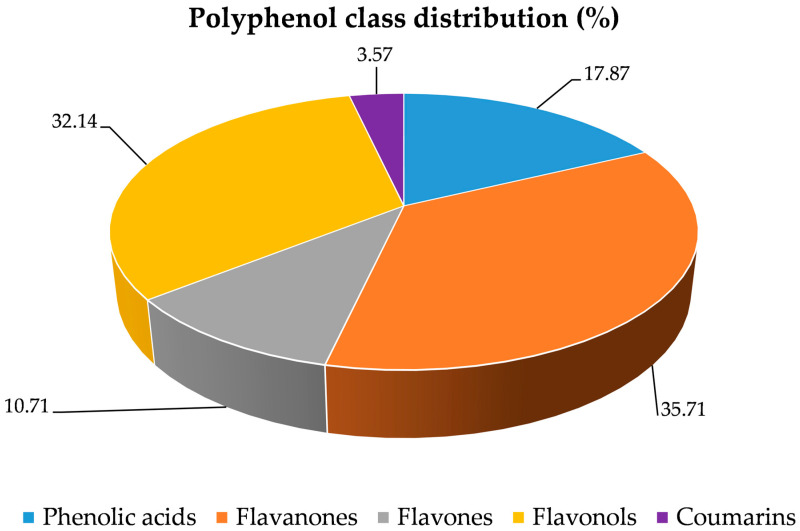
Classification of the 28 polyphenolic constituents identified in *C. lumia* ‘Pyriformis’ by LC–MS/MS. The pie chart illustrates their distribution across the main polyphenolic classes (phenolic acids, flavanones, flavones, flavonols, coumarins, and others), providing an overview of the chemical composition.

The specific quantification of flavan-3-ols, determined through the vanillin assay, yielded 6.09 ± 0.25 mg CE/100 mL, while the proanthocyanidin content, expressed as cyanidin chloride equivalents, was 0.76 ± 0.03 mg/100 g. The polymerization index, calculated as the ratio between the vanillin index and the proanthocyanidin content, was 8.07, indicating a predominance of low-condensed oligomeric forms. The polyphenolic profile of CLPJ, analyzed by RP-LC-DAD-ESI-MS/MS, led to the identification of 28 phenolic compounds belonging to the main structural classes of phenolic acids, flavones, flavonols, flavanones, and coumarins (Table 3 and Figure 1). Among phenolic acids, 3,4-dicaffeoylquinic acid and caffeoyl-feruloylquinic acid were detected in trace amounts. The flavonoid fraction was characterized by the presence of glycosides of naringenin, narirutin, hesperetin, isorhamnetin, and quercetin, confirming the occurrence of multiple flavonoid subclasses. Naringenin-7-*O*-glucoside exhibited a peak intensity of 1.69 × 10^6^, while neohesperidin, one of the most abundant flavanone glycosides, showed the highest peak intensity (8.99 × 10^6^). Among flavonols, myricetin 3-*O*-galactoside and quercetin-*O*-(malonyl-glucoside) were the most represented, with peak intensities of 7.85 × 10^6^ and 2.99 × 10^6^, respectively. Quercetin-(hydroxybenzoyl-coumaroyl rhamnoside) was also identified, although at lower intensity. The presence of tricin-*O*-[glucuronopyranosyl-*O*-methyl-*O*-glucuronopyranoside], with a peak intensity of 2.68 × 10^6^, was also recorded. Acylated glycosylated derivatives such as hesperetin-*O*-(feruloyl-rutinoside) and hesperetin-*O*-(feruloyl-glucosyl-rutinoside) were detected, together with the coumarin bergaptol, identified at low concentration. Notably, the compounds with the highest peak intensities overall were the flavonol isorhamnetin-*O*-glucuronopyranosyl-*O*-glucuronopyranoside (10.80 × 10^6^), the methylated flavonol limocitrol-*O*-rutinoside (10.57 × 10^6^), and the dihydrochalcone neohesperidin dihydrochalcone (10.29 × 10^6^). Their abundance further highlights the complex glycosylation and acylation patterns present in CLPJ and underlines the contribution of flavonol and flavanone derivatives to the polyphenolic profile.

Finally, it is worth noting that the phytochemical characterization of the polyphenolic profile is fully consistent with the results of the preliminary phytochemical screening (Table 1), confirming the substantial presence of total phenols, flavonoids, and flavan-3-ols, thereby reinforcing the analytical reliability and coherence of the entire phenolic profile of the juice.

**Figure 2:**
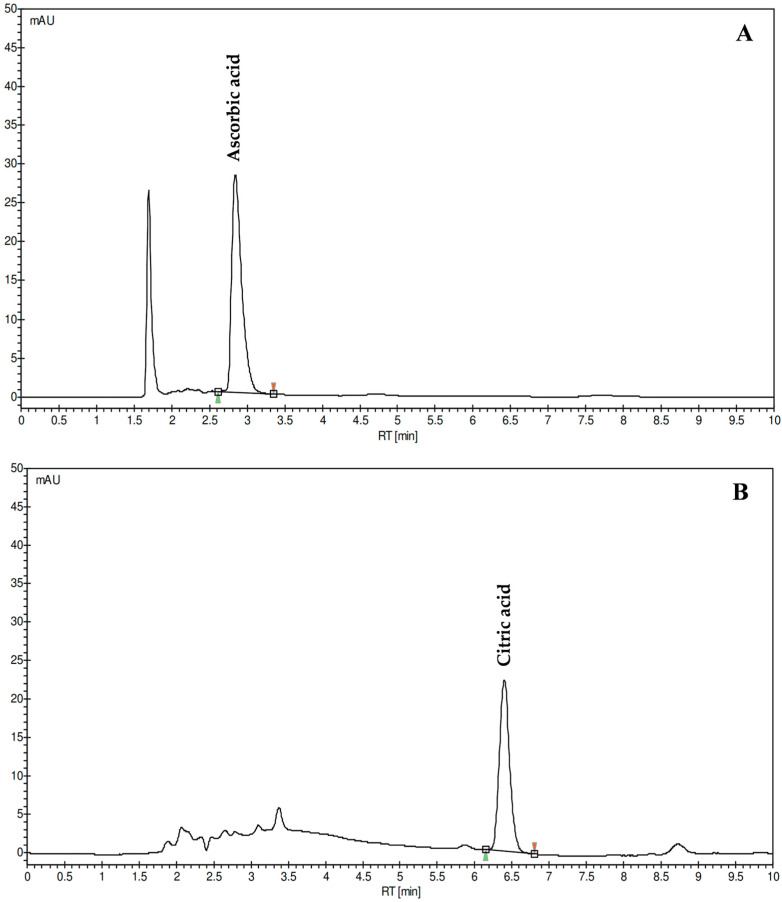
Representative chromatograms of ascorbic acid (**A**) and citric acid (**B**) identified and quantified in *C. lumia* ‘Pyriformis’ juice (CLPJ; 12 mg/mL and 5 mg/mL, respectively) by HPLC-DAD. Chromatograms were acquired at 245 nm and 210 nm for ascorbic and citric acid, respectively.

The quantification of ascorbic acid and citric acid in CLPJ was performed by HPLC-DAD analysis at compound-specific wavelengths (Figure 2).

Both analytes were clearly resolved, with sharp and symmetrical chromatographic peaks and no detectable interferences. The concentration of ascorbic acid was 0.34 g/L, corresponding to a substantial content of this water-soluble vitamin in the analyzed juice. The citric acid concentration was 34.64 g/L, representing the major organic acid component of the matrix. The chromatographic method allowed precise quantification of both compounds, which are known to contribute significantly to the chemical composition, redox balance, and acidity of *Citrus* juices.

Overall, the results obtained depict a well-defined phytochemical and organic acid profile for CLPJ, characterized by a balanced distribution of phenolic compounds, flavonoids, flavan-3-ols, and organic acids. The coexistence of these molecules indicates a complex biochemical composition that forms the basis for the subsequent evaluation of its antioxidant, anti-inflammatory, and wound-healing activities.

### 3.2. Biological Properties

The antioxidant activity of CLPJ was evaluated using a panel of in vitro assays based on different mechanisms of action, including electron transfer, hydrogen atom transfer, and reducing capacity, in both hydrophilic and lipophilic systems. The corresponding IC_50_ values, together with their 95% confidence limits, are reported in Table 4.

CLPJ showed measurable efficacy in the DPPH, TEAC, and FRAP assays, all representative of electron-transfer mechanisms in the aqueous phase and exhibited the highest activity in the ORAC and BCB assays, which simulated peroxyl radical neutralization and lipid oxidation, respectively. In contrast, no detectable activity was observed in the ICA assay. The latter result is probably attributable to the high content of citric acid (34.64 g/L), which forms stable Fe^2+^–citrate complexes that interfere with ferrozine binding and thus with spectrophotometric detection at 562 nm. No pH adjustment or acid removal was performed to maintain the native conditions of the matrix.

The anti-inflammatory potential of CLPJ was assessed through the inhibition of protein denaturation (ADA) and proteolytic activity (PIA). In both assays, CLPJ showed concentration-dependent inhibitory effects, although with lower potency compared with the pharmacological reference, sodium diclofenac, which was tested in the microgram range. However, the results indicate that CLPJ possesses measurable activity against protein denaturation and trypsin-mediated casein hydrolysis (Table 4).

**Figure 3:**
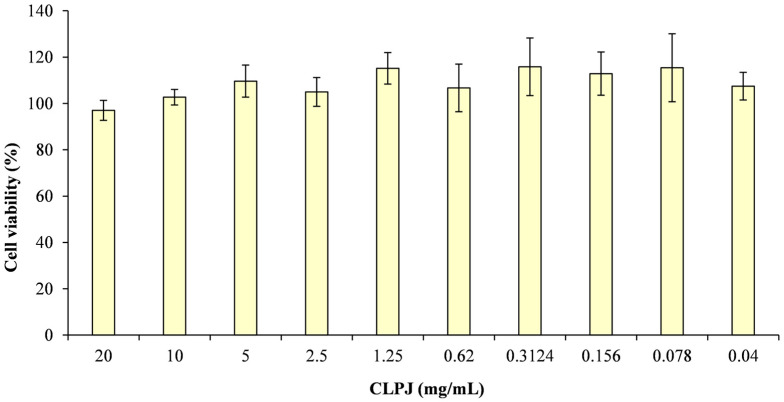
Determination of cell viability (%) at 48 h by MTT assay following treatment of Caco-2 cells with different concentrations of *C. lumia* ‘Pyriformis’ juice (CLPJ).

Cytotoxicity was evaluated using the MTT assay on Caco-2 intestinal epithelial cells after 24 and 48 h of exposure. As no statistically significant differences were found between the two time points, only the 48 h results are reported (Figure 3).

Across the tested concentration range (0.04–20 mg/mL), CLPJ did not induce any cytotoxic effects. Mean cell viability values remained between 97% and 115% of the untreated control, without statistically significant variations among concentrations. Even at the highest tested concentrations (10 and 20 mg/mL), mitochondrial metabolic activity was comparable to that of the control, confirming the absence of acute toxicity under the tested conditions.

Based on these findings, five concentrations (0.782–12.5 mg/mL) were selected for the wound-healing assay. The concentration of 20 mg/mL was excluded because, despite not showing significant cytotoxicity, it produced a minor reduction in cell viability. The wound-healing test was performed on confluent Caco-2 monolayers to evaluate the capacity of CLPJ to promote epithelial restitution under biocompatible conditions.

In Caco-2 cells, *Citrus* flavonoids have been previously shown to modulate oxidative stress and inflammatory mediators, supporting the type of epithelial responses observed in the present model [12].

**Figure 4:**
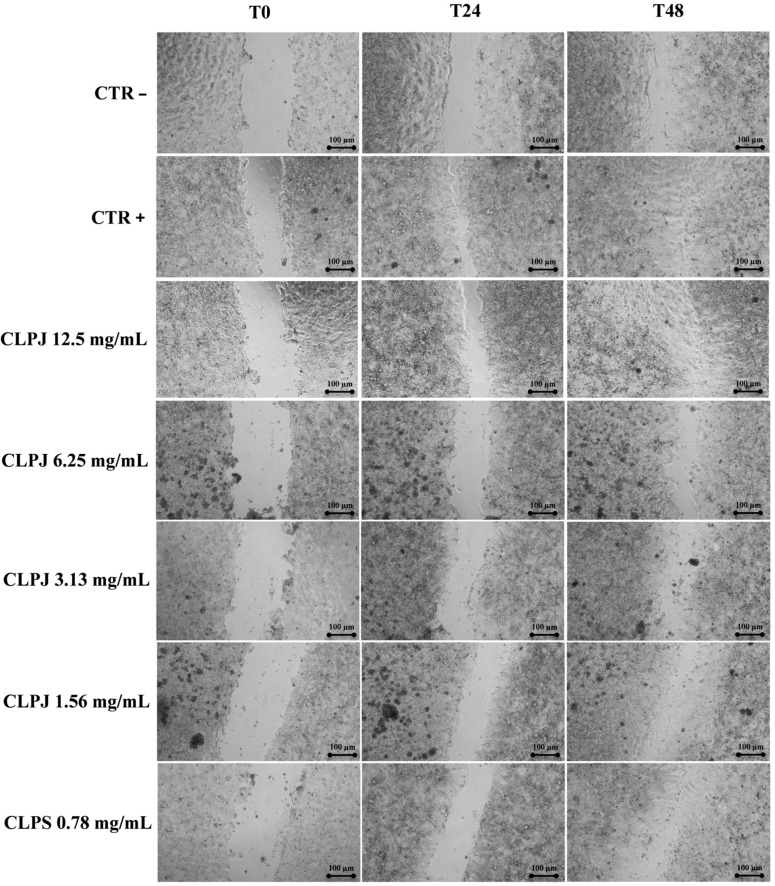
Wound-healing assay on Caco-2 cells to assess cell migration following treatment. Wound closure was evaluated at T_0_, and after 24 h and 48 h of incubation. CTR–: complete DMEM (negative control); CTR+: 0.1% hyaluronic acid in complete DMEM (positive control). The juice was diluted in complete DMEM. Scale bar: 100 µm.

**Figure 5:**
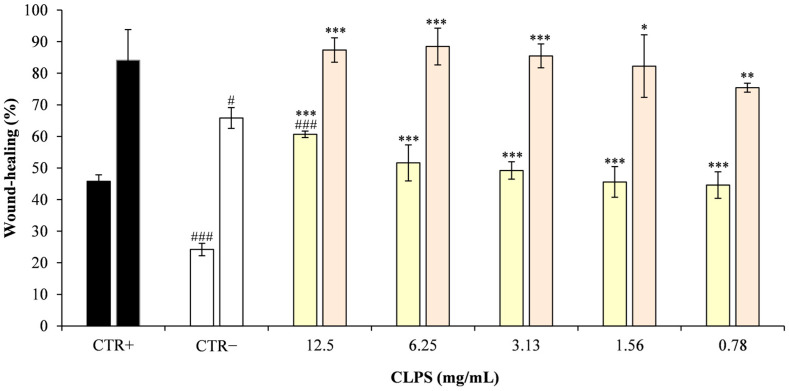
Wound-healing activity (%) in Caco-2 cells. For each treatment condition, the first bar represents the percentage of wound closure at 24 h, and the second bar represents the corresponding value at 48 h. Data are expressed as the mean ± SD of three independent experiments performed in triplicate and calculated relative to time zero (T_0_). Statistical significance was assessed by comparing each treatment with both the negative control (CTR−) and the positive control (CTR+, 0.1% hyaluronic acid). Asterisks indicate significant differences versus CTR− (* *p* < 0.05, ** *p* < 0.01, *** *p* < 0.001), whereas hash symbols indicate significant differences versus CTR+ (^#^ *p* < 0.05, ^###^ *p* < 0.001).

As shown in Figure 4 and Figure 5, CLPJ promoted wound closure in a concentration-dependent manner. After 24 h, wound closure values were 60.7% ± 1.0 at 12.5 mg/mL, 51.6% ± 5.7 at 6.25 mg/mL, and 49.2% ± 2.7 at 3.125 mg/mL, compared with 45.8% ± 2.0 for the positive control (CTR+) and 24.2% ± 1.95 for the negative control (CTR−). After 48 h, further progression was observed, with wound closure of 87.4% ± 3.8 at 12.5 mg/mL, 88.5% ± 5.8 at 6.25 mg/mL, 85.5% ± 3.8 at 3.125 mg/mL, and 75.4% ± 1.4 at 0.782 mg/mL, compared with 65.8% ± 3.3 for the CTR−.

Statistical analysis revealed that CLPJ at 3.13, 6.25, and 12.5 mg/mL significantly increased wound closure compared with the CTR− at both 24 and 48 h (*p* < 0.001). At 48 h, the 1.56 mg/mL and 0.782 mg/mL concentrations also produced significant improvements vs. CTR− (*p* < 0.05 and *p* < 0.01, respectively), while at 24 h both concentrations were already significantly effective at *p* < 0.001, in line with the higher doses.

In addition, while the highest concentration (12.5 mg/mL) showed a significantly greater effect than the CTR+ at 24 h, at 48 h all tested concentrations displayed wound-healing activity comparable to the CTR+, with no statistically significant differences.

The results indicate that CLPJ effectively enhanced wound closure in Caco-2 monolayers in a concentration-dependent manner. Within the limits of the experimental design, this activity can be ascribed to increased cell migration, although a contribution from cell proliferation cannot be excluded, as no proliferation inhibitor was applied.

Overall, CLPJ demonstrated multi-mechanistic antioxidant and anti-inflammatory activities, absence of cytotoxicity, and a concentration-dependent capacity to promote epithelial wound closure under in vitro conditions consistent with intestinal exposure.

## 4. Discussion

The preliminary phytochemical screening of fresh *C. lumia* Risso & Poit. ‘Pyriformis’ juice (CLPJ) revealed a matrix rich in flavonoids and phenolic acids. The total phenolic content falls within the range reported for several *Citrus* juices—*C. aurantifolia*, *C. medica*, *C. aurantium*, *C. nobilis*, *C. ichangensis*, and *C. karna* (≈28–75 mg/100 mL), with *C. limetta* being the only species showing a markedly higher value (≈117 mg/100 mL) [17]. In contrast, *C. lumia* juice exhibits much higher concentrations (≈819 mg/100 mL) [12], comparable only to polyphenol-enriched *Citrus* extracts, which cannot be directly compared to native juices [18].

CLPJ showed a modest amount of proanthocyanidins (condensed tannins). However, under the low pH conditions typical of *Citrus* matrices, particularly those with high citric acid content, proanthocyanidins may undergo depolymerization and degradation, leading to reduced redox capacity and analytical detectability [19]. Considering the high citric acid concentration measured in CLPJ (34.64 g/L), the relatively low level of these compounds may be partially explained by pH-dependent instability. Consequently, the redox activity observed in vitro likely arises mainly from phenolic subclasses that are more stable in acidic environments, such as glycosylated flavanones, flavonols, and phenolic acids.

Comprehensive RP-LC-DAD-ESI-MS/MS analysis identified 28 phenolic compounds belonging to phenolic acids, flavones, flavonols, flavanones, and coumarins. The profile was characterized by abundant glycosylated flavanones (e.g., neohesperidin, neohesperidin dihydrochalcone, hesperetin derivatives), rare flavonols (e.g., quercetin-*O*-(-malonyl-glucoside), myricetin 3-*O*-galactoside), and complex structures such as tricin-*O*-[glucuronopyranosyl-*O*-methyl-*O*-glucuronopyranoside]. Compared with *C. sinensis*, *C. paradisi*, *C. reticulata* and *C. clementina*, which generally display simpler polyphenolic profiles with fewer acyl-glycosylated flavones/flavonols [20,21], CLPJ exhibited higher structural diversity and greater representation of flavonols. Several features overlap with *C. lumia* [12] including the presence of quinic acid derivatives and characteristic flavonoids such as naringenin-7-*O*-glucoside and isorhamnetin-*O*-diglucuronide, while the ‘Pyriformis’ variety shows additional diversification with acyl-glycosidic derivatives such as hesperetin-*O*-(feruloyl-rutinoside) and hesperetin-*O*-(feruloyl-glucosyl-rutinoside). The presence of methylated and acylated flavanones mirrors that observed in *C. bergamia* [22], suggesting a partial phytochemical overlap between these two species.

Importantly, the detection of uncommon flavonols (e.g., quercetin-*O*-(malonyl-glucoside)), highly acylated flavanones, and complex *O*-/C-glycosylated flavone derivatives provides chemotaxonomic evidence that distinguishes *C. lumia* ‘Pyriformis’ from commercial sweet *Citrus* species [23,24]. The structural pattern observed—characterized by acyl glycosides, rare flavonols, and multi-glycosylated conjugates—is more consistent with wild or primitive *Citrus* lineages than with cultivated lemons or sweet oranges, underscoring the unique phytochemical identity of this minor taxon.

Analysis of organic acids revealed high levels of citric acid and ascorbic acid. Reported citric acid contents in *Citrus* juices vary widely—from ≈74 g/L in lemon (*C. limon*) and ≈62 g/L in lime (*C. aurantifolia*) to much lower concentrations in sweet orange (*C. sinensis*, ≈13.9 g/L), clementine (*C. clementina*, ≈11.9 g/L), and mandarin (*C. reticulata*, ≈8.9 g/L) [25]. The value for CLPJ therefore lies in an intermediate range, higher than in most sweet *Citrus* species but lower than in lemon and lime. This pronounced acidity contributes to both the sensory and chemical characteristics of the juice. Moreover, citric acid acts as a natural chelating agent for divalent metals, which likely explains the absence of measurable chelating activity in the ferrozine assay, where Fe^2+^–citrate complexation prevents chromophore formation. This behavior represents an analytical limitation rather than a true absence of metal-binding potential under physiological conditions. Ascorbic acid levels in CLPJ are comparable to those in lemon, lime, and *C. lumia*, and exceed those typically found in sweet orange, clementine, and grapefruit [12,25]. Since ascorbic acid is one of the major hydrophilic antioxidants in *Citrus* juices—capable of both direct radical scavenging and regeneration of oxidized antioxidants—its abundance likely contributes synergistically with phenolic compounds to the overall redox capacity of the matrix.

The antioxidant and anti-inflammatory properties of CLPJ were evaluated through a series of complementary in vitro assays encompassing multiple mechanisms (electron transfer, hydrogen atom transfer, and reducing power) and across different environments. CLPJ showed measurable activity in DPPH, TEAC, and FRAP assays, and higher efficacy in ORAC and β-carotene bleaching, confirming its ability to counteract both hydrophilic and lipophilic oxidative processes. No activity was detected in the iron-chelating test under native acidic conditions, in agreement with the citric acid–Fe^2+^ interaction previously discussed.

The results obtained place CLPJ within the intermediate range of antioxidant capacity among *Citrus* juices [17], reflecting a compositional balance dominated by stable glycosylated flavonoids and acyl-glycosides. Comparisons with the dataset reported by Wang et al. [18] show that, when normalized as mg Trolox equivalents per g of juice, CLPJ displays values of the same order of magnitude as those of polyphenol-enriched extracts for DPPH and ORAC, and slightly lower for FRAP, despite being a native, non-purified matrix. This supports the hypothesis of synergistic interactions among naturally co-occurring phytochemical classes.

In the Caco-2 cell model, CLPJ showed no cytotoxicity up to 20 mg/mL after 24 and 48 h, confirming its excellent biocompatibility. The absence of cytotoxic effects allowed the evaluation of its pro-reparative capacity through a wound-healing assay, which demonstrated a significant and concentration-dependent enhancement of wound closure after 24 and 48 h. At 24 h, the highest concentration (12.5 mg/mL) induced significantly greater wound closure than both the negative and positive controls, whereas at 48 h, all tested concentrations showed activity comparable to the positive control. Given the lack of a proliferation inhibitor, a contribution from cell proliferation cannot be entirely excluded; however, the constant viability values between 24 and 48 h suggest a predominant effect on cell migration and epithelial restitution.

This behavior aligns with the known biological properties of *Citrus* flavonoids such as hesperetin, hesperidin, and naringenin, which modulate oxidative and inflammatory signaling pathways [26,27,28]. Hesperetin has been associated with SIRT3-mediated protection against oxidative stress and ferroptosis, mechanisms essential for tissue repair. Hesperidin has been shown to accelerate gastric mucosal healing by enhancing antioxidant defenses and reducing inflammation, while naringenin has demonstrated wound-healing activity through inhibition of AKT/MAPK/NF-κB signaling and reduction in ROS and pro-inflammatory cytokines. The phytochemical composition of CLPJ, which includes these compounds and related glycosides, plausibly underlies the pro-migratory effect observed in the Caco-2 monolayer. Although direct studies assessing wound-healing behavior in Caco-2 monolayers are scarce, available investigations demonstrate that these cells respond to redox-active and anti-inflammatory phytochemicals in a manner consistent with the effects observed for CLPJ [29].

Comparable evidence has been reported for other *Citrus*-derived matrices, such as *C. hystrix* extract, which enhanced wound closure in keratinocytes and fibroblasts through antioxidant and anti-inflammatory mechanisms [29]. Although based on different epithelial systems, these findings support the concept that polyphenol-rich *Citrus* juices and extracts can promote epithelial repair. In our case, the use of fresh, unprocessed juice provides an added advantage, as it preserves the native molecular complexity and synergistic interactions among phytochemicals.

Overall, CLPJ emerges as a distinctive *Citrus* matrix characterized by high citric acidity, substantial vitamin C content, and a complex phenolic composition. It exhibits multi-assay antioxidant and anti-inflammatory activity, excellent cytocompatibility, and a marked capacity to promote epithelial wound closure in vitro. These findings support its potential application as a nutraceutical ingredient or functional food component aimed at protecting and restoring epithelial integrity, warranting further mechanistic and translational investigation.

However, this study is based on juice obtained from a single plant source, which may not fully reflect the natural phytochemical variability of *C. lumia* ‘Pyriformis’. Seasonal, environmental, and agronomic factors could influence the metabolite composition and should be considered in future sampling campaigns. Although the LC-MS/MS profiling provides a detailed qualitative characterization, confirmation through broader metabolomic approaches and additional samples would strengthen the chemotaxonomic interpretation. Likewise, the in vitro biological effects observed here warrant validation in more complex models, including intestinal explants and in vivo systems, to better assess their physiological relevance.

## 5. Conclusions

This study demonstrates the fresh juice of *C. lumia* Risso & Poit. var. ‘Pyriformis’ possesses a distinctive phytochemical signature, characterized by a rich diversity of glycosylated flavonoids, phenolic acids, and redox-active compounds such as ascorbic and citric acids. Although the total polyphenol content and antioxidant capacity are lower than those of the progenitor *C. lumia*, CLPJ exhibits a highly diversified polyphenolic profile and relevant biological activities. The combined antioxidant, anti-inflammatory, and epithelial reparative properties highlight its potential as a functional food ingredient for maintaining intestinal integrity. Future investigations employing advanced in vitro models should clarify the molecular pathways involved and validate its efficacy under physiologically relevant conditions. Overall, CLPJ represents a promising example of the nutraceutical valorization of minor *Citrus* varieties within the framework of sustainable and clean-label food innovation, providing new insights into the health-promoting potential of Mediterranean *Citrus* biodiversity.

## Figures and Tables

**Table 1:** Analytical validation of the HPLC-DAD method for the determination of ascorbic and citric acids, including linearity, regression parameters, sensitivity (LOD/LOQ), precision, and recovery values.

Parameter	Ascorbic Acid	Citric Acid
Calibration range (µg/mL)	0.3125–10	12.5–200
Equation	y = 0.9543x	y = 0.0203x
R^2^	0.999	0.999
LOD (µg/mL)	0.12	0.36
LOQ (µg/mL)	3.0	9.0
Recovery (%)	98.5	99.0

**Table 2:** Phytochemical screening of *C. lumia* Risso & Poit. ‘Pyriformis’ juice (CLPJ). Results are expressed as the mean ± standard deviation of three independent experiments performed in triplicate (*n* = 3).

Phytochemical Assay	CLPJ
Total phenols (mg GAE ^a^/100 mL)	38.80 ± 0.99
Total flavonoids (mg RE ^b^/100 mL)	25.96 ± 2.37
Vanillin index (mg CE ^c^/100 mL)	6.09 ± 0.25
Proanthocyanidins (mg CcE ^d^/100 mL)	0.76 ± 0.03
Polymerization index ^e^	8.07

^a^ GAE, gallic acid equivalents; ^b^ RE, rutin equivalents; ^c^ CE, catechin equivalents; ^d^ CcE, cyanidin chloride equivalents; ^e^ Vanillin index/Proanthocyanidins ratio.

**Table 3:** LC-DAD-ESI-MS/MS phytochemical characterization of *C. lumia* Risso & Poit. ‘Pyriformis’ juice (CLPJ). Peak intensity refers to the maximum ion signal (peak height) recorded in the Total Ion Current (TIC) chromatogram for each compound. The colored dots indicate the corresponding class, as shown in Figure 1.

Compound	Class	RT ^a^ (min)	MW ^b^	[M + H]^+^ (*m*/*z*)	MS/MS (*m*/*z*)	[M − H]^−^ (*m*/*z*)	MS/MS (*m*/*z*)	PI ^c^ (×10^6^)
Limocitrol-*O*-rutinoside	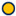	1.7	538	539	301,393	537	391,299	10.57
3,4-Dicaffeoylquinic acid ^d^	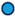	2.6	516	–	–	515	353,191,179	0.76
Narirutin-*O*-glucoside ^d^	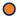	4.6	772	773	611,465,303	771	609,463,301	0.34
Naringin-4-*O*-glucoside ^d^	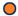	5.6	742	743	581,435,273	741	579,433,271	1.01
Dicaffeoylshikimic acid	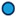	13.1	510	511	355,193	509	353,191,179,135	1.23
Apigenin-*O*-deoxy-hexosyl-hexoside	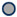	13.6	578	579	433,271,153	577	431,269,151	0.12
Quercetin-*O*-(*O*-malonylglucosyl-rhamnoside)	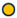	17.0	712	713	647,303	711	627,565,463,301	2.99
Naringenin-7-*O*-glucoside ^d^	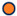	20.9	434	435	273,153,147	433	271,151,119	1.69
Myricetin 3-*O*-galactoside ^d^	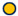	22.9	480	481	319,165,153	479	317,179,151	7.85
Apigenin-*C*-hexoside-*O*-pentoside	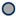	23.0	564	565	435,313,271	563	473,433,383,353,341	0.20
Quercetin-*O*-(malonyl-glucoside)-*O*-glucoside	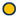	25.9	712	713	549,487,303	711	625,549,487,301,179	1.17
Quercetin-*O*-deoxyhexosylhexoside-*O*-hexoside	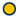	26.2	772	773	611,465, 303	771	625,609,463,301,179	0.13
Caffeoyl-feruloylquinic acid	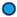	26.9	530	–	–	529	353,369,191,179,149	1.09
Neoeriocitrin ^d^	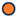	27.9	596	597	451,289,271	595	449,287	0.17
Limocitrol-*O*-hydroxy-3-methylglutaryl-hexoside	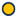	29.5	652	653	509,347,329	651	507,343,313	0.14
Hesperetin-*O*-(feruloyl-rutinoside)	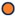	30.1	786	787	611,465,303,195	785	609,463,301,193	1.36
Sinapoyl-feruloylgentiobioside	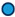	32.3	724	725	531,501,307,225,195	723	529,499,305,223,193	2.90
Tricin-*O*-[glucuronopyranosyl-*O*-methyl-*O*-glucuronopyranoside]	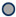	32.9	696	697	521,501,333,193,177	695	519,499,331,175,151	2.68
Bergaptol	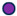	34.3	202	203	175,147,119	201	173,159,119	0.45
Hesperetin-*O*-(feruloyl-glucosyl-rutinoside)	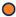	34.9	974	975	799,637,491,303,195	973	797,635,489,301,193,149	1.24
Quercetin-(p-hydroxybenzoyl-p-coumaroyl rhamnoside)	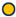	37.4	714	–	593,569,303,165,123	713	591,567,445,299,163,121	0.14
Neohesperidin ^d^	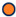	37.7	610	611	465,303	609	463,301,151	8.99
Isorhamnetin-*O*-glucuronopyranosyl-*O*-glucuronopyranoside	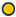	38.3	668	669	493,317,195	667	491,315,193,149	10.80
Naringenin-*O*-deoxyhexosylhexoside-*O*-hexoside	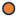	38.4	742	743	597,435,273,147	741	595,433,271,163	0.14
Hesperetin ^d^	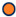	55.8	302	303	288	301	286	4.39
(E)-3-(3,4-Diacetoxy-5-methoxyphenyl)-acryloyl-p-coumaroyl-caffeoylquinic acid	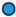	65.6	777	778	616,598,537,355,181,165	776	614,596,535,354,179,163	0.08
Neohesperidin dihydrochalcone	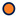	72.3	612	613	467,305,147	611	465,303,163	10.29
Kaempferol-*O*-triglucoside	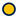	79.7	772	773	611,449,287	771	609,447,285	6.18

^a^ RT, retention time; ^b^ MW, molecular weight; ^c^ PI, peak intensity; ^d^ confirmed with commercially available HPLC-grade reference standards (purity ≥ 98%) purchased from Extrasynthase (Genay, France) or Merck KGaA (Darmstadt, Germany).

**Table 4:** Antioxidant and anti-inflammatory properties of *C. lumia* Risso & Poit. ‘Pyriformis’ juice (CLPJ). Results are expressed as the concentration of juice required to inhibit 50% of the oxidative or inflammatory activity (IC_50_), with the corresponding 95% confidence limits in parentheses. Data represent the mean ± standard deviation of three independent experiments performed in triplicate (*n* = 3).

Test	CLPJ (IC_50_ mg/mL)	Reference Compound ^a^ (IC_50_ µg/mL)
DPPH	15.62 (12.15–20.09)	7.80 (2.85–21.40)
FRAP	8.0 (6.05–10.73)	3.64 (1.32–10.03)
TEAC	11.58 (2.23–16.26)	5.32 (2.86–9.88)
ORAC	1.06 (0.51–2.20)	0.61 (0.3–0.95)
BCB	0.8 (0.49–1.32)	0.44 (0.21–0.81)
ICA	n.a.	4,08 (2.78–5.88)
ADA	0.82 (0.16–4.25)	16.87 (13.61–20.92)
PIA	5.57 (4.59–6.76)	25.79 (14.87–44.74)

^a^ Trolox for DPPH, FRAP, TEAC, and ORAC; EDTA for ICA; BHT for BCB; and sodium diclofenac for anti-inflammatory assays ADA and PIA. n.a. = not active.

## Data Availability

The original contributions presented in this study are included in the article. Further inquiries can be directed to the corresponding author.

## Abbreviations

The following abbreviations are used in this manuscript:

AAPH	2,2′-Azobis(2-methylpropionamidine) Dihydrochloride
ABTS	2,2′-Azino-bis-(3-ethylbenzothiazoline-6-sulfonic acid)
ADA	Albumin Denaturation Assay
ANOVA	Analysis of Variance
BCB	β-Carotene Bleaching
BHT	Butylated Hydroxytoluene
BSA	Bovine Serum Albumin
*C. lumia*	*Citrus lumia* Risso & Poit.
CcE	Cyanidin Chloride Equivalents
CE	Catechin Equivalents
CH_3_COONa	Sodium Acetate
CLPJ	*Citrus lumia* ‘Pyriformis’ juice
CO_2_	Carbon Dioxide
DMEM	Dulbecco’s Modified Eagle’s Medium
DMSO	Dimethyl Sulfoxide
DPPH	2,2-Diphenyl-1-picrylhydrazyl
EDTA	Ethylenediaminetetraacetic Acid
FBS	Fetal Bovine Serum
FeSO_4_·7H_2_O	Ferrous Sulfate Heptahydrate
FRAP	Ferric Reducing Antioxidant Power
GAE	Gallic Acid Equivalents
HPLC-DAD	High-Performance Liquid Chromatography with Diode Array Detection
IC_50_	Half Maximal Inhibitory Concentration
ICA	Iron-Chelating Activity
KH_2_PO_4_	Potassium Dihydrogen Phosphate
LC-DAD-ESI-MS/MS	Liquid Chromatography–Diode Array Detection–Electrospray Ionization–Tandem Mass Spectrometry
Na_2_CO_3_	Sodium Carbonate
Na_2_HPO_4_	Disodium Hydrogen Phosphate
NaOH	Sodium Hydroxide
NEAA	Non-Essential Amino Acids
ORAC	Oxygen Radical Absorbance Capacity
PBS	Phosphate-Buffered Saline
PEN/STREP	Penicillin–Streptomycin
PIA	Protease Inhibition Assay
RE	Rutin Equivalents
RT	Room Temperature
SD	Standard Deviation
TEAC	Trolox Equivalent Antioxidant Capacity
TPTZ	2,4,6-Tris(2-pyridyl)-s-triazine
UV–Vis	Ultraviolet–Visible
